# Supplementary figures and images for: Accucopy: accurate and fast inference of allele-specific copy number alterations from low-coverage low-purity tumor sequencing data
Source: BMC Bioinformatics. 2021 Jan 15;22:23. doi: 10.1186/s12859-020-03924-5 (PMC7811225; doi:10.1186/s12859-020-03924-5)

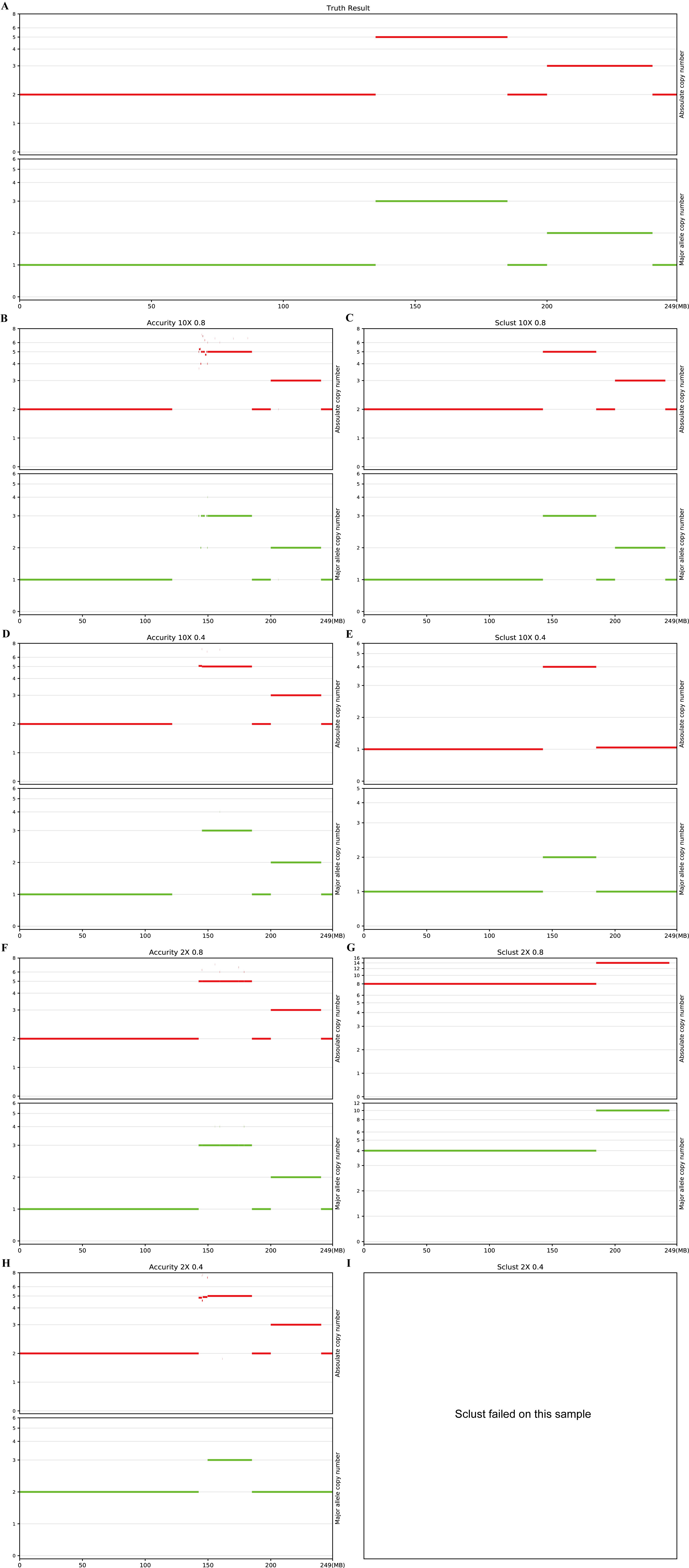

Supplement: Supplementary file 1 — Additional file 1. Supplementary Fig. 1: Copy number profile of chr1 on partial simulation data. X-axis is the chromosomal position and Y-axis is the copy number. Each figure has two panels. The top is the profile of absolute copy number and the bottom is the profile of major allele copy number. A. The truth profile of chr1. B-I. The copy number profile given by different methods on different samples. The title of each figure has three keys split by space, which indicate the method name, sample coverage and sample purity respectively. [file 12859_2020_3924_MOESM1_ESM.jpeg]
