# Supplementary material for: Accucopy: accurate and fast inference of allele-specific copy number alterations from low-coverage low-purity tumor sequencing data
Source: BMC Bioinformatics. 2021 Jan 15;22:23. doi: 10.1186/s12859-020-03924-5 (PMC7811225; doi:10.1186/s12859-020-03924-5)
Supplement: Supplementary file 2 — Additional file 2. Method details of Accucopy. [file 12859_2020_3924_MOESM2_ESM.docx]

**Additional File 1 Method details of Accucopy**

1. **Basic definitions**

We define the fraction of cancer cells in a tumor sample as the tumor purity γ and the fraction of normal cells is 1-γ. We assume that the ploidy of a normal cell is 2 and denote the average copy number of a cancer cell as the tumor cell ploidy: κ. The tumor sample ploidy ω is a weighted average of the ploidy of normal and cancer cells, expressed in γ and κ as follows:

| $\begin{aligned} \omega=\left( 1-\gamma\right)\times2+\gamma\times\kappa\end{aligned}$ | (1) |
| --- | --- |

We denote the total copy number (TCN) of a chromosomal segment s of all tumor cells as $C_{s}$. Then, the TCN of the same segment for the tumor sample, $C_{t}$, is the average TCN of tumor and normal cells in the tumor sample:

| $\begin{aligned} C_{t}=\left( 1-\gamma\right)\times2+\gamma\times C_{s} \end{aligned}$ | (2) |
| --- | --- |

Note the difference between the tumor cell ploidy and the tumor sample ploidy. The latter includes ploidy contribution from normal cells in a tumor sample while the former is only about tumor cells. The two are identical for a 100% pure tumor. Similarly, the tumor cell TCN of a segment is different from the tumor sample TCN of the same segment. The observed sequencing coverage of a tumor sample should be proportional to the tumor sample ploidy, thus dependent on the tumor purity and tumor cell ploidy, $\gamma, \kappa$.

1. **Tumor Read Enrichment (TRE) for a chromosomal segment**

Denote the number of reads covering a genomic segment s for a tumor sample and its matching normal sample as $n_{t}^{s}$ and $n_{n}^{s}$, respectively, and a total number of $N_{t}$ and $N_{n}$ reads for a tumor sample and its matching normal sample. The Tumor Read Enrichment (TRE) for segment bin s, $e_{s}$, is defined as follows:

| $\begin{aligned} e_{s}={\frac{n_{t}^{s}}{N_{t}}}/{\frac{n_{n}^{s}}{N_{n}}} \end{aligned}$ | (3) |
| --- | --- |

TRE is a normalized read enrichment of a chromosomal segment in a tumor sample relative to its matching normal sample. Factors that influence both tumor and normal samples, such as the read mappability and the GC biases, are canceled out. To have a better statistical representation, TRE is calculated for each 500bp (roughly the sequencing fragment length) bin throughout the whole genome. A fast version of GADA is applied to segment the entire genome based on calculated TREs.

1. **The TRE expectation and the TCN Gaussian mixture model**

For a chromosomal segment bin s, assuming independence between the local and global coverage, the expected TRE of a segment bin s can be approximated as follows:

| $\begin{aligned} E_{s}=E\left( e_{s} \right)=E\left( {\frac{n_{t}^{s}}{N_{t}}}/{\frac{n_{n}^{s}}{N_{n}}} \right)\approx\frac{E\left( n_{t}^{s} \right)}{E\left( n_{n}^{s} \right)}\times\frac{E\left( N_{n} \right)}{E\left( N_{t} \right)} \end{aligned}$ | (4) |
| --- | --- |

We define a few nuisance parameters to help to further derive $E_{s}$. The length of segment bin $s$ is $L_{s}$. The length of the reference genome, about three billions, is $L_{\mathrm{gw}}$. The genome-wide average sequencing coverage is $V_{\mathrm{gw}}^{T}$ for the tumor sample and $V_{\mathrm{gw}}^{N}$ for its matching normal sample. The average sequencing coverage for segment bin $s$ from a tumor sample is $\lambda_{s}\times V_{\mathrm{gw}}^{T}$, which multiplies a sequence-specific factor $\lambda_{s}$ to the genome-wide sequencing coverage. The average sequencing coverage for segment bin $s$ from the matching normal sample is $\lambda_{s}\times V_{\mathrm{gw}}^{N}$. With all these definitions, we can derive the expected TRE, $E_{s}$, as a statistic only dependent on tumor purity, $\gamma$, tumor cell ploidy, $\kappa$, and the TCN of the segment bin in cancer cell, $C_{s}$:

| $\begin{aligned} E_{s}=\frac{E\left( n_{t}^{s} \right)}{E\left( n_{n}^{s} \right)}\times\frac{E\left( N_{n} \right)}{E\left( N_{t} \right)}=\frac{C_{t}\times L_{s}\times\lambda_{s}\times V_{\mathrm{gw}}^{T}}{2\times L_{s}\times\lambda_{s}\times V_{\mathrm{gw}}^{N}}\times\frac{2\times L_{\mathrm{gw}}\times V_{\mathrm{gw}}^{N}}{\omega\times L_{\mathrm{gw}}\times V_{\mathrm{gw}}^{T}} \\ =\frac{C_{t}}{\omega}=\frac{\left( 1-\gamma\right)\times2+\gamma\times C_{s}}{\left( 1-\gamma\right)\times2+\gamma\times\kappa} \end{aligned}$ | (5) |
| --- | --- |

The entire segment, s, is assumed to be of the same TCN and thus all observed $e_{s}$ should have the same expectation, $E_{s}$. Thus, we drop the subscript $s$ of $E_{s}$ and add the superscript $i$ to denote the expected TRE for all segments with $\text{TCN}=i$ as $E^{i}$:

| $\begin{aligned} E^{i}=\frac{\left( 1-\gamma\right)\times2+\gamma\times i}{\left( 1-\gamma\right)\times2+\gamma\times\kappa} \end{aligned}$ | (6) |
| --- | --- |

For all segments with TCN=$i+1$, the corresponding $E^{i+1}$ is

| $\begin{aligned} E^{i+1}=\frac{\left( 1-\gamma\right)\times2+\gamma\times\left( i+1 \right)}{\left( 1-\gamma\right)\times2+\gamma\times\kappa} \end{aligned}$ | (7) |
| --- | --- |

Forms of $E^{i}$ and $E^{i+1}$ can explain the periodicity we observed from any TRE histogram. We define the period of a TRE histogram, $P$, as the interval between two copy numbers (**Figure 1**) and its expected value is

**Figure 1 A typical Tumor Read Enrichment (TRE) histogram shows a periodic pattern**

Auto-correlation analysis can identify the period of the histogram, P, as the interval between major peaks. Q, one major peak that corresponds to copy-number-two segments, is identified through the Accucopy probabilistic model. Minor peaks between the major ones consist of subclonal segments.


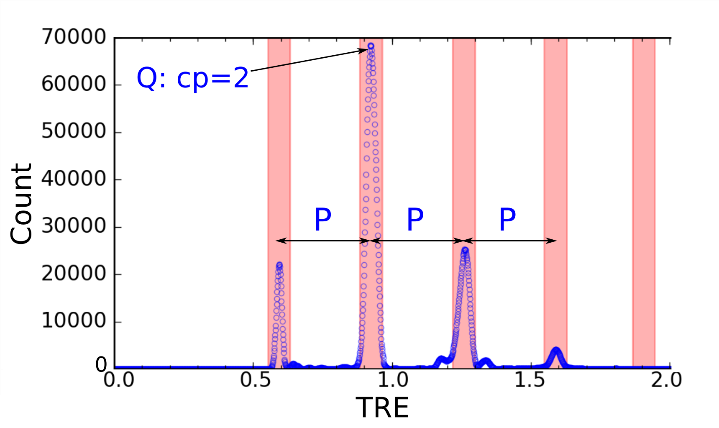


| $\begin{aligned} P=E^{i+1}-E^{i}=\frac{\gamma}{\left( 1-\gamma\right)\times2+\gamma\times\kappa} \end{aligned}$ | (8) |
| --- | --- |

In a histogram of TREs (Figure 1), the period $P$ is the interval between two adjacent major peaks. Each major peak in a TRE histogram represents one group of clonal segments with the same integral copy number. Usually the period of a tumor sample decreases with low purity or high ploidy.

Further, we define the Normal TRE (NTRE) $Q$, as the TRE corresponding to segments of copy number 2, then

| $\begin{aligned} Q &=E^{i}\vert\left( i=2 \right)=\frac{\left( 1-\gamma\right)\times2+\gamma\times i}{\left( 1-\gamma\right)\times2+\gamma\times\kappa}\vert\left( i=2 \right) \\ &=\frac{2}{\left( 1-\gamma\right)\times2+\gamma\times\kappa} \end{aligned}$ | (9) |
| --- | --- |

Solving eq. 8 and 9 produces the tumor sample purity $\gamma$ and the tumor cell ploidy $\kappa$ in terms of $P$ and $Q$.

| $\begin{aligned} \gamma=\frac{2\times P}{Q} \\ \kappa=2+\frac{1-Q}{P} \end{aligned}$ | (10) |
| --- | --- |

We model the observed TREs, $e_{s}$, as a Gaussian mixture with each component corresponding to TCN $=i$.

| $\begin{aligned} e_{s}\sim\Sigma_{i=0}^{I}p_{i}N\left( E^{i}, \sigma_{i}^{2} \right) \end{aligned}$ | (11) |
| --- | --- |

where $p_{i}$ is the mixing parameter (also prior probability) for component $i$, with $\Sigma_{i=0}^{I}p_{i}=1$, and $\sigma_{i}^{2}$ is the component variance. The TRE likelihood for all M segments is:

| $\begin{aligned} L\left( e;\gamma,\kappa\right)=\prod_{s=1}^{M} \sum_{i=0}^{I} p_{i}P(e_{s}\vert E^{i},\sigma_{i}^{2}) \end{aligned}$ | (12) |
| --- | --- |

**4 Log ratio of Allelic-coverage Ratios (LAR) of HGSNVs**

For an HGSNV, denote $n_{t}^{R}$, $n_{t}^{A}$, $n_{n}^{R}$ and $n_{n}^{A}$ as the read counts for the reference allele (R) and the alternative allele (A) in a tumor (t) and its matching normal (n) samples. Define $r$ as the log-ratio of allelic-coverage ratios (LAR) for an HGSNV:

| $\begin{aligned} r=\log\left( \left( \frac{n_{t}^{R}}{n_{n}^{R}} \right)/\left( \frac{n_{t}^{A}}{n_{n}^{A}} \right) \right)=log(\frac{n_{t}^{R}n_{n}^{A}}{n_{n}^{R}n_{t}^{A}}) \end{aligned}$ | (13) |
| --- | --- |

The LAR is defined in the same vein as the TRE. The tumor allelic coverage is normalized by that of the matching normal sample to eliminate various sequencing biases: the GC-bias, the reference mapping bias, etc. However, the definition requires all four read counts to be positive as any zero will render the statistic ill-defined, which necessitates an adjustment in calculating its expectation, detailed in the next section.

The variant calling of HGSNVs is carried out at 44 million SNP loci from the 1000 Genomes project using Strelka2. To improve the quality of the final HGSNVs, the tumor and normal samples were called simultaneously by Strelka2, in so-called multi-sample calling, SNPs must be heterozygous in the normal sample, and the coverage of the SNP must be above two in either sample.

**5 The LAR expectation, the ASCN Gaussian mixture model, and the EM algorithm**

Denote the ASCNs of a tumor cell and its matching normal cell at an HGSNV as $(k,l)$, lower case of L in it, and $(1,1)$, with $k$ and $l$ denoting the major-allele copy number and the minor-allele copy number in the tumor cell respectively. The alternative allele is less likely to be mapped correctly to the reference genome than the reference allele due to the reference bias. Let $\phi$ denote the reference mapping bias of the reference allele relative to the alternative allele, and typically $\phi>1$. Hence, the ASCNs of a pure tumor sample and its matching normal one is either $(\phi k,l)$ and $(\phi,1)$, or $(k,\phi l)$ and $(1,\phi)$, depending on if the major allele is the reference allele or not. Taking the tumor purity $\gamma$ into account, the ASCNs of a tumor sample is either $(\phi(\gamma k+\left( 1-\gamma\right)\times1), \gamma l+\left( 1-\gamma\right)\times1)$, or $(\gamma k+\left( 1-\gamma\right)\times1, \phi(\gamma l+\left( 1-\gamma\right)\times1))$, depending on which allele is the reference allele, with the ASCNs of the normal sample unchanged. The sequencing coverage of a segment is proportional to its copy number. Then take expectation of eq. 13 produces the following naïve expectations of LAR, with the reference bias $\phi$ cancelled out:

| $\begin{aligned} E\left( r \right)=\mu_{1}^{*} or \mu_{2}^{*} \\ \mu_{1}^{*}=\log\frac{\gamma k+\left( 1-\gamma\right)\times1}{\gamma l+\left( 1-\gamma\right)\times1}\text{ } \\ \mu_{2}^{*}=\log\frac{\gamma l+\left( 1-\gamma\right)\times1}{\gamma k+\left( 1-\gamma\right)\times1}\# \end{aligned}$ | (14) |
| --- | --- |
| $\begin{aligned} \mu_{1}^{*}=-\mu_{2}^{*} \end{aligned}$ | (15) |

However, the definition of LAR (eq. 13) precludes HGSNVs with any zero allelic coverage in either sample, which creates a substantial bias not accounted for in the naïve expectation (eq. 14), we model the allelic sequencing coverage as a Poisson distribution and exclude zero-coverage to derive a better expectation of LAR. Denote $\lambda_{k+l}=\lambda_{k}+\lambda_{l}$ as the mean total coverage, with$\lambda_{k}$ and $\lambda_{l}$ being the mean coverage of the major and minor alleles respectively:

| $\begin{aligned} \lambda_{k}=\frac{\gamma k+\left( 1-\gamma\right)}{\gamma(k+l)+2\times\left( 1-\gamma\right)}\times\lambda_{k+l} \end{aligned}$ | (16) |
| --- | --- |
| $\begin{aligned} \lambda_{l}=\frac{\gamma l+\left( 1-\gamma\right)}{\gamma(k+l)+2\times\left( 1-\gamma\right)}\times\lambda_{k+l} \end{aligned}$ | (17) |

We estimate $\lambda_{k+l}$ as the median depth of all HGSNVs within a segment in a tumor sample. Denote $d^{k}$ and $d^{l}$ as the observed read counts of the major and the minor alleles respectively and each follows a Poisson distribution:

| $\begin{aligned} d^{k}\sim Po\left( \lambda_{k} \right), d^{l}\sim Po\left( \lambda_{l} \right) \end{aligned}$ | (18) |
| --- | --- |

Excluding the zero read counts, the adjusted expectation of LAR, $\mu_{1}$ and $\mu_{2}$, are as follows:

| $\begin{aligned} \mu_{1}=\frac{\sum_{d^{k}=1}^{\infty} \sum_{d^{l}=1}^{\infty} \log\left( \frac{d^{k}}{d^{l}} \right)P\left( d^{k}\vert\lambda_{k} \right)P\left( d^{l}\vert\lambda_{l} \right)}{\sum_{d^{k}=1}^{\infty} \sum_{d^{l}=1}^{\infty} P\left( d^{k}\vert\lambda_{k} \right)P\left( d^{l}\vert\lambda_{l} \right)} \\ \mu_{2}= -\mu_{1} \end{aligned}$ | (19) |
| --- | --- |

**Figure 2** shows the adjustment greatly improved the fit between the observed mean LAR and the true mean.

**Figure 2 Effect of the adjustment of the expectation of LAR for a segment of HCC1187**

This is a histogram of LARs for this segment. The known TCN (Total Copy Number) of this segment is 3. The known ASCN (major allele vs minor allele) is 3-0. The gray bars are the observations. The blue line is the fitted Gaussian mixture distribution based on the naïve expectations of LAR. The red line is the fitted Gaussian mixture distribution based on the adjusted expectations of LAR.


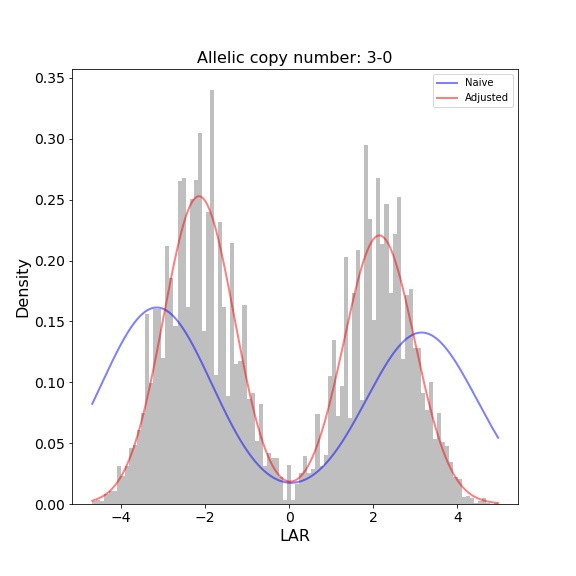


In real data, we have no idea if the expectation of an observed LAR is $\mu_{1}$or $\mu_{2}$because it is unknown which allele is the major allele. Thus, we adopt a two-component Gaussian mixture model with the two components having an identical variance and their means opposite to each other:

| $\begin{aligned} r\sim\Sigma_{m=1}^{2}\alpha_{m}N\left( \mu_{m}, \sigma_{s}^{2} \right) \end{aligned}$ | (20) |
| --- | --- |

where $\alpha_{m}$ is the mixing parameter (or prior probability) for component m, with $\alpha_{1}+\alpha_{2}=1$, and $\sigma_{s}^{2}$ is the Gaussian variance of LAR for either component, specific to segment s.

We introduce a missing variable, $\Delta$, that indicates which component an LAR belongs to, and apply the Expectation-Maximization (EM) algorithm to estimate $\alpha_{m}$ and $\sigma_{s}^{2}$. Given a segment s with TCN = $C_{s}$, which contains $N^{s}$ LARs, for every possible ASCN combination $\left( k,l \right):k=C_{s}-l, l=0,1,\cdots,\left\lceil C_{s}/2 \right\rceil$, $\mu_{m}$ is calculated according to eq. 19, then the E-step computes the conditional probability of one LAR belonging to either component:

| $\begin{aligned} P\left( \Delta_{i}\vert r_{i};\sigma_{s}^{2\left( g \right)},\alpha_{1}^{\left( g \right)},\alpha_{2}^{\left( g \right)} \right)=\frac{\alpha_{m}^{\left( g \right)}P\left( r_{i}\vert\mu_{m},\sigma_{s}^{2\left( g \right)} \right)}{\sum_{m=1}^{2} \alpha_{m}^{\left( g \right)}P\left( r_{i}\vert\mu_{m},\sigma_{s}^{2\left( g \right)} \right)} \\ \Delta_{i}=1,2; i=1,2,\ldots, N_{s} \end{aligned}$ | (21) |
| --- | --- |

The M-step updates these parameters:

| $\begin{aligned} &\hat{\sigma}_{s}^{2}=\frac{1}{N_{s}}\sum_{i=1}^{N_{s}} \sum_{\Delta_{i}=1,2} P\left( \Delta_{i}\vert r_{i},\sigma_{s}^{2\left( g \right)},\alpha_{1}^{\left( g \right)},\alpha_{2}^{\left( g \right)} \right)\left( r_{i}-\mu_{m} \right)^{2} \\ &\hat{\alpha}_{1}=\frac{1}{N_{s}}\sum_{i=1}^{N_{s}} P\left( \Delta_{i}=1\vert r_{i},\sigma_{s}^{2\left( g \right)},\alpha_{1}^{\left( g \right)},\alpha_{2}^{\left( g \right)} \right) \\ &\hat{\alpha}_{2}=1-\hat{\alpha}_{1}^{\left( g \right)} \end{aligned}$ | (22) |
| --- | --- |

The E-step and M-step are iterated until convergence and we calculate the LAR likelihood for ASCN $(k,l)$ as follows:

| $\begin{aligned} L\left( r;k,l \right)=\prod_{i=1}^{N_{s}} \sum_{m=1,2} \alpha_{m}P(r_{i}\vert\mu_{m}, \sigma_{s}^{2}) \end{aligned}$ | (23) |
| --- | --- |

The EM algorithm is applied to solve optimal parameters and derive the corresponding likelihood for every possible ASCN combination $\left( k,l \right)$. The ASCN estimate $\left( \hat{k},\hat{l} \right)$ for segment $s$ is the one with the maximum likelihood.

| $\begin{aligned} \left( \hat{k},\hat{l} \right)=\arg\max_{k,l} \log L\left( r;k,l \right) \end{aligned}$ | (24) |
| --- | --- |

In the next step, the maximum LAR likelihood of all segments will be combined with the TRE likelihood via the Bayesian Information Criterion (BIC) to determine the most likely tumor purity, tumor ploidy, TCNs and ASCNs of all chromosomal segments.

**6 BIC for the combined model and optimization**

To avoid model overfitting, we adopt the Bayesian Information Criterion (BIC).

| $\begin{aligned} BIC\left( e,r;\gamma,\kappa,k,l \right)=-2\log L\left( e;\gamma,\kappa\right)-2\log L\left( r;k,l \right) \\ +I\times\log M+J\times\log N \end{aligned}$ | (25) |
| --- | --- |

where I is the total number of potential TCNs, M is the total number of observed TREs, J is the total number of potential ASCNs, and $N$ is the total number of HGSNVs.

Instead of searching through the infinite range of tumor purity $\gamma\in\left[ 0,1 \right]$, the tumor cell ploidy $\kappa\in\left[ 0,\infty\right]$, all possible TCNs and ASCNs, an optimization scheme that leverages the periodic TRE pattern is adopted. Accucopy first uses an autocorrelation analysis that discovers candidates for P and Q, which are equivalent to $\gamma$ and $\kappa$, as shown in eq. 10, and finds the minimum BIC score among these candidates only.

To reduce the noise in the TRE distribution, Accucopy applies a kernel smoothing (1D Gaussian) before the autocorrelation analysis. The top two lags identified in the auto-correlation analysis form the candidates of $P$. Given a candidate $P$, Accucopy further identifies major peaks in the TRE distribution that are $P$ apart, which represent clonal segments of integral copy numbers, and filters out segments that do not belong to any major peak, which are classified as subclonal segments. The TREs of all the major peaks become the candidates for $Q$. Given a pair of candidate $P$ and $Q$, the TCN of each clonal segment is determined, the TRE likelihood is computed, and the EM algorithm is carried out to compute the most likely ASCN of this segment and its LAR likelihood. The TRE and LAR likelihoods are then combined into the BIC score. The best estimates of purity and ploidy $\left( \hat{\gamma},\hat{\kappa} \right)$, TCNs, and ASCNs $(\hat{k},\hat{l})$ are obtained by minimizing the BIC score:

| $\begin{aligned} \left( \hat{\gamma},\hat{\kappa}, \hat{k},\hat{l} \right)=\arg\min_{\gamma,\kappa,\mu,k,l} BIC\left( e,r;\gamma,\kappa,k,l \right) \end{aligned}$ | (26) |
| --- | --- |
